# p63 Transcription Factor Regulates Nuclear Shape and Expression of Nuclear Envelope-Associated Genes in Epidermal Keratinocytes

**DOI:** 10.1016/j.jid.2017.05.013

**Published:** 2017-10

**Authors:** Valentina Rapisarda, Igor Malashchuk, Inemo E. Asamaowei, Krzysztof Poterlowicz, Michael Y. Fessing, Andrey A. Sharov, Iakowos Karakesisoglou, Vladimir A. Botchkarev, Andrei Mardaryev

**Affiliations:** 1 Centre for Skin Sciences, University of Bradford, Bradford, UK; 2 Department of Dermatology, Boston University School of Medicine, Boston, Massachusetts, USA; 3 Department of Biosciences, University of Durham, Durham, UK

**Keywords:** CC, chromocenter, ChIP-qPCR, chromatin immunoprecipitation-quantitative PCR, H3K9me3, trimethylation on lysine 9 of histone H3, H3K27me3, trimethylation on lysine 27 of histone H3, KtyI, keratin type I, KtyII, keratin type II, PMK, primary mouse keratinocyte, IF, intermediate filament, NM, nuclear membrane, NE, nuclear envelope, WT, wild-type

## Abstract

The maintenance of a proper nuclear architecture and three-dimensional organization of the genes, enhancer elements, and transcription machinery plays an essential role in tissue development and regeneration. Here we show that in the developing skin, epidermal progenitor cells of mice lacking p63 transcription factor display alterations in the nuclear shape accompanied by a marked decrease in expression of several nuclear envelope-associated components (Lamin B1, Lamin A/C, Sun1, Nesprin-3, Plectin) compared with controls. Furthermore, chromatin immunoprecipitation-quantitative PCR assay showed enrichment of p63 on *Sun1*, *Syne3*, and *Plec* promoters, suggesting them as p63 targets. Alterations in the nuclei shape and expression of nuclear envelope-associated proteins were accompanied by altered distribution patterns of the repressive histone marks trimethylation on lysine 27 of histone H3, trimethylation on lysine 9 of histone H3, and heterochromatin protein 1-alpha in p63-null keratinocytes. These changes were also accompanied by downregulation of the transcriptional activity and relocation of the keratinocyte-specific gene loci away from the sites of active transcription toward the heterochromatin-enriched repressive nuclear compartments in p63-null cells. These data demonstrate functional links between the nuclear envelope organization, chromatin architecture, and gene expression in keratinocytes and suggest nuclear envelope-associated genes as important targets mediating p63-regulated gene expression program in the epidermis.

## Introduction

Epidermis is a stratified self-renewing epithelium, in which lineage-committed progenitor cells residing in the basal layer proliferate and differentiate into cells of the suprabasal layers to form epidermal barrier (Blanpain and Fuchs, 2009, Fuchs, 2007). Terminal differentiation in epidermal keratinocytes is accompanied by structural and biochemical changes in the nucleus associated with its transition from a highly active state in the basal layer to fully inactive state in the cornified layer, where DNA is degraded and nucleus is eliminated (Botchkarev et al., 2012, Eckhart et al., 2013). Differentiating epidermal keratinocytes markedly change their nuclear shape and three-dimensional (3D) nuclear organization, including spatial relationships between pericentromeric heterochromatin clusters, nucleoli, and chromosome territories (Gdula et al., 2013).

Nuclear shape and size are controlled by the nuclear envelope (NE) that provides anchoring sites for several cytoskeletal components and chromatin at the outer and inner nuclear membranes (NMs), respectively. The NE plays a crucial role in regulating the mechanical stability of the nucleus, nucleocytoplasmic transport, chromatin organization, and gene expression (Hetzer, 2010, Kim et al., 2015, Wilson and Foisner, 2010). Proteins of the linker of nucleoskeleton and cytoskeleton complex (such as nesprins-1/2/3/4) interact directly with the cytoplasmic cytoskeleton on the outer NM. At the inner NM, a different set of linker of nucleoskeleton and cytoskeleton proteins, such as Sun1/2, interact with nuclear lamins, thus forming “bridges” that link outer and inner membranes and establish physical connections between the cytoskeleton and chromatin (Hetzer, 2010, Sosa et al., 2013).

In keratinocytes, both keratin filaments and nuclear lamins contribute to the regulation of nuclear shape and integrity. Cytokeratin 14 filaments form a cage-like perinuclear structure, which is required for resizing and reshaping of nuclei in early differentiating keratinocytes, whereas *Krt14* gene ablation results in alterations of nuclear shape in epidermal keratinocytes (Lee et al., 2012, Troy and Turksen, 1999). In addition, keratin 1/10 deletion decreases expression of NE-associated proteins, such as emerin, lamin A/C, and Sun1, leading to premature nuclei loss during epidermal differentiation (Wallace et al., 2012).

Nuclear lamins (Lamin A/C, Lamin B1, and Lamin B2) are intermediate filaments (IFs) forming an interconnected meshwork (lamina) underlying the inner NM. They also contribute to the regulation of nuclear shape and link inner NM to the chromatin via interaction with its lamina-associated domains (Hetzer, 2010, Kind et al., 2015). Loss of Lamin A/C and Lamin B receptors leads to the loss of peripheral heterochromatin in many cell types including hair follicle keratinocytes (Solovei et al., 2013). Moreover, genetic ablation of all three nuclear lamins in keratinocytes resulted in the development of ichthyosis and skin barrier defects (Jung et al., 2014).

p63 transcription factor is a master regulator of epidermal development as its deletion leads to profound defects in epidermal morphogenesis (Mills et al., 1999, Yang et al., 1999). p63 controls expression of a large number of genes controlling cell adhesion, signaling, and lineage-specific components of the cytoskeleton, such as keratins (Kouwenhoven et al., 2010, Kouwenhoven et al., 2015, McDade et al., 2012, Viganò et al., 2006, Zarnegar et al., 2012). We also reported that p63 controls expression of a number of chromatin remodelers, such as Satb1, Brg1, and Cbx4, that coordinate gene expression in epidermal progenitor cells during development (Fessing et al., 2011, Mardaryev et al., 2014, Mardaryev et al., 2016). In this report, we show that p63 regulates the nuclear shape and expression of NE-associated genes, coupled to changes in heterochromatin organization and intranuclear position of keratin loci in keratinocytes. These data suggest a complex role for p63 in the integration of cytoskeleton, NE, and chromatin remodeling factors in epidermal progenitor cell differentiation during morphogenesis.

## Results

### Skin epithelial cells in p63 knockout mice display nuclear shape alterations

**Figure 1:**
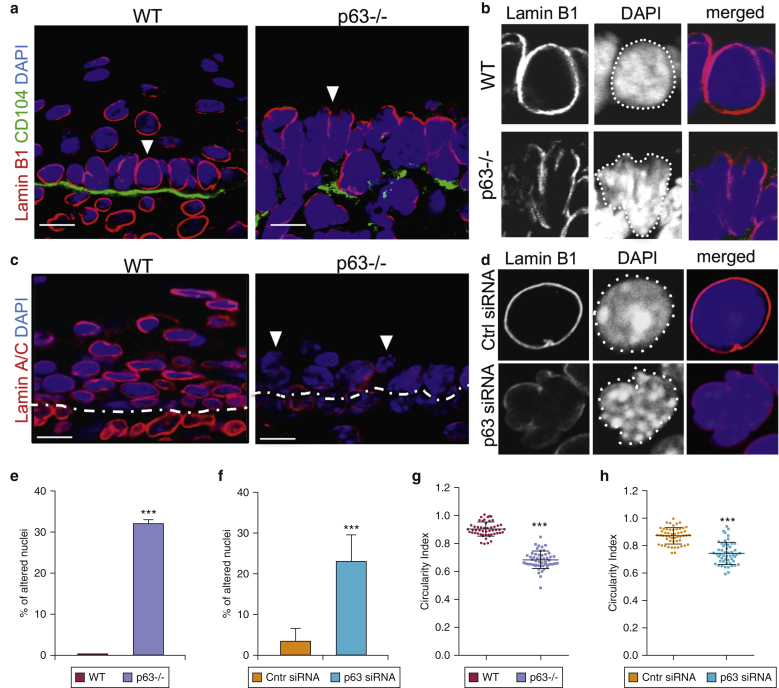
**In vivo and in vitro analysis of WT and p63-null keratinocytes nuclear shape.** (**a, b**) In vivo analysis of the nuclear shape of basal keratinocytes in p63-null embryos versus wild-type controls, stained with Lamin B1. CD104 (Integrin b4) staining depicts the basement membrane (**a**). Arrowheads (**a**) indicate nuclei shown enlarged (**b**). Scale bars = 10 μm. (**c**) Lamin A/C expression in *p63*^*−/−*^ keratinocytes. Note a decreased expression of Lamin A/C in *p63*^*−/−*^ keratinocytes with altered nuclear shape (arrowheads). The dashed line delineates the dermal-epidermal junction. Scale bars = 10 μm. (**d**) Decreased expression of Lamin B1 and altered nuclear shape in primary mouse keratinocytes transfected with p63 siRNA. (**e, f**) Quantification of keratinocytes with altered nuclear shape in (**e**) *p63*^*−/−*^ mice and (**f**) p63-depleted keratinocytes in vitro. Chi-square test (mean ± SD, ***P-value < 0.001). (**g, h**) Quantification of the nuclear circularity index in (**g**) *p63*^*−/−*^ mice and (**h**) p63-depleted keratinocytes in vitro. Student’s *t*-test (mean ± SD, ***P-value < 0.001). SD, standard deviation; siRNA, small interfering RNA; WT, wild-type.

Emerging data suggest that the cytoskeleton plays an important role in nuclear morphology and chromatin organization (Li et al., 2014, Ramdas and Shivashankar, 2015, Xue and Funabiki, 2014). As several p63 targets encode cytoskeletal components, we speculated that p63 may regulate nuclear morphology. To test this, we analyzed expression of NE-associated proteins in E16.5 *p63*^*−/−*^ embryos and age-matched wild-type (WT) controls. Immunostaining with anti-Lamin B1 and anti-Lamin A/C antibodies revealed an epidermal-specific decrease in expressions of nuclear lamins in *p63*^*−/−*^ mice compared with controls, whereas dermal cells were not affected (Figure 1a–c; Supplementary Figure S1a and b online). Interestingly, the reduced nuclear lamin expressions were more profound in cells with abnormal nuclear shape in *p63*^*−/−*^ keratinocytes (Figure 1a–c, arrowheads). Lamin B1 expression was also reduced in p63-depleted primary mouse keratinocytes (PMKs) transfected with p63-specific siRNA (Figure 1d; Supplementary Figure S2a and b online).

To characterize the nuclear shape changes on p63 deletion, we measured a nuclear circularity index, which defines alterations and variations in the nuclear shape based on how closely each nucleus corresponds to a spherical shape (a perfect sphere has a value of 1). In agreement with previous reports, we considered nuclei with a circularity index <0.8 as altered in shape (Ray and Chapman, 2015, Schochlin et al., 2014). Our analysis revealed that approximately 32% of p63-null keratinocytes had abnormal nuclei with circularity index <0.8 (compared with 0.5% of cells in WT controls) (Figure 1e and g). Furthermore, p63 knockdown in PMKs revealed a significant increase in the number of cells (23%) with altered nuclear shape compared with only 3% of control cells (Figure 1f and h). The nuclear shape changes were keratinocyte specific, as the circularity index was not significantly altered in *p63*^*−/−*^ dermal cells compared with controls (Supplementary Figure S1b and c).

As changes in the nuclear shape can be caused by apoptosis (Rao et al., 1996, Raz et al., 2006), we analyzed the expression of active caspase-3 in *p63*^*−/−*^ mice. All epidermal keratinocytes with misshapen nuclei were negative for the caspase-3 (Supplementary Figure S3a online), indicating that alterations in the nuclear shape were associated with mechanisms other than apoptosis in *p63*^*−/−*^ mice. Consistently with this observation, most cells with misshapen nuclei were actively proliferating as determined by Ki-67 staining (Supplementary Figure S3b).

### Decreased expression of NE-associated proteins in p63-null keratinocytes

**Figure 2:**
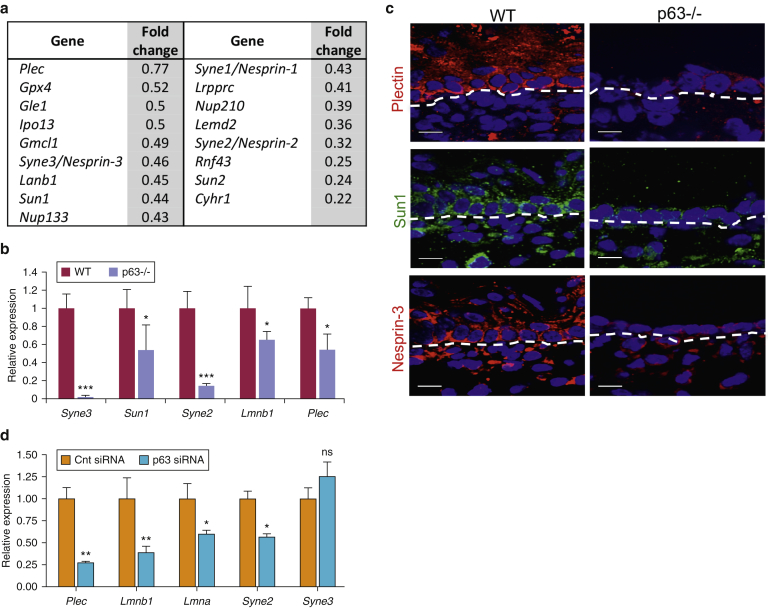
**Nuclear envelope-associated proteins are reduced in p63-null keratinocytes.** (**a**) Agilent microarray data demonstrating changes in expression of genes encoding nuclear envelope-associated proteins between *p63*^*−/−*^ and WT mice. (**b**) Real-time PCR validation of the microarray analysis for *Plec*, *Sun1*, *Syne3*, *Syne2* and *Lmnb1* in the E16.5 epidermis of p63^−/−^ mice normalized to the corresponding levels in the age-matched WT epidermis (mean ± SD, n = 3, ∗*P* < 0.05, ∗∗∗*P* < 0.001). (**c**) Immunostaining for plectin, SUN1 and nesprin-3 in the E16.5 skin of WT and p63^−/−^ mice. Scale bar, 10 μm. Dashed lines depict dermal-epidermal junction. (**d**) Real-time PCR analysis for *Plec*, *Sun1*, *Syne2/3, Lmna*, and *Lmnb1* expression in PMK after p63 knockdown using siRNA (mean ± SD, n = 3, *P < 0.05, **P < 0.001). PMK, primary mouse keratinocyte; SD, standard deviation; WT, wild-type.

Because epidermal cells in *p63*^*−/−*^ mice and p63-depleted PMKs showed a marked reduction in Lamin B1 and Lamin A/C (Figure 1a–d), we asked if other NE-associated proteins were also affected in p63-null epidermis. The analysis of our previously published *p63*^*−/−*^ transcriptome (Fessing et al., 2011, Mardaryev et al., 2014) revealed a downregulation of 17 transcripts encoding NE and NE-associated proteins in p63-null epidermis compared with controls (Figure 2a).

To validate the microarray data, we selected a list of genes (*Plec, Sun1*, *Syne2*, *Syne3*, and *Lmnb1*) for further analysis. We found a significant decrease in expression of the selected genes by quantitative real-time reverse transcriptase–PCR in *p63*^*−/−*^ keratinocytes versus WT controls (Figure 2b). Immunostaining also confirmed the reduced expression of Plectin, Sun1, and Nesprin-3 proteins in the *p63*^*−/−*^ epidermis compared with controls (Figure 2c). Furthermore, siRNA-mediated p63 knockdown revealed a significant decrease in expression of *Lmnb1*, *Lmna*, *Plec*, *Sun1*, *Syne2* but not *Syne3* in PMKs (Figure 2d).

### p63 binds to the regulatory regions of the *Plec*, *Sun1*, and *Syne3* genes

**Figure 3:**
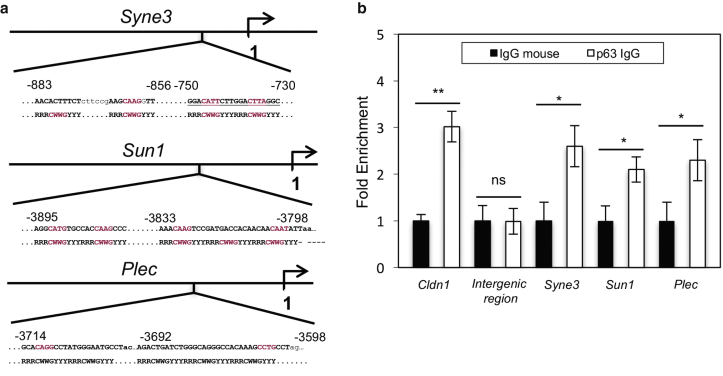
**p63 is enriched at the promoter regions of the *Syne3*, *Sun1*, and *Plec* genes.** Chromatin isolated from primary mouse keratinocytes was processed for ChIP assay with an antibody against p63 protein or purified mouse IgG. (**a**) Regions within the promoter of *Syne3, Sun1*, and *Plec* analyzed by ChIP-qPCR. Matching p63 core binding site consensus sequences are in red. (**b**) Enrichment of p63 at the *Syne3*, *Sun1*, and *Plec* promoter regions. The input levels of unprecipitated chromatin DNA were used as loading controls. *Cldn1* and an intergenic region on chr. 8 were used as positive and negative controls, respectively. Error bars represent SD, and four independent experiments were run in triplicates; *P* < 0.05. ChIP-qPCR, chromatin immunoprecipitation-quantitative PCR; SD, standard deviation.

To test whether the *Sun1, Syne3*, *Plec*, and *Lmnb1* genes may be direct p63 targets in keratinocytes, we performed chromatin immunoprecipitation with anti-p63 antibody followed by quantitative PCR (ChIP-qPCR) analysis in PMKs isolated from newborn WT mice. Using a PatSearch tool (Grillo et al., 2003), we designed qPCR primers to multiple sites within a 6-kb-long region upstream to transcription start sites, containing several putative p63-binding sites (Figure 3a). ChIP-qPCR revealed p63 binding to *Plec, Sun1*, and *Syne3* within the analyzed regions (Figure 3b), suggesting that all three genes are direct p63 targets in mice. In contrast, we did not see any p63 enrichment on *Lmnb1* even by testing multiple predicted sites within the *Lmnb1* promoter region (Supplementary Figure S4a online), suggesting an indirect regulation by p63. To test if the same genes targeted by p63 in normal human epidermal keratinocytes, we reanalyzed the publicly available p63 ChIP-seq dataset and ENCODE data for enhancer-specific histone modifications (H3K4me1, H3K27ac) (ENCODE Project Consortium, 2012, Kouwenhoven et al., 2010). Similar to mice, p63 was coenriched with the histone marks in the promoter and proximal enhancer regions (up to 10 kb from transcription start sites) of *PLEC*, *SUN1*, and *SYNE3* in normal human epidermal keratinocytes. However, p63 did not bind to *LMNB1* promoter or distant enhancers even within 50 kb from the transcription start sites (Supplementary Figure S4b).

### p63-null keratinocytes show an altered heterochromatin organization

**Figure 4:**
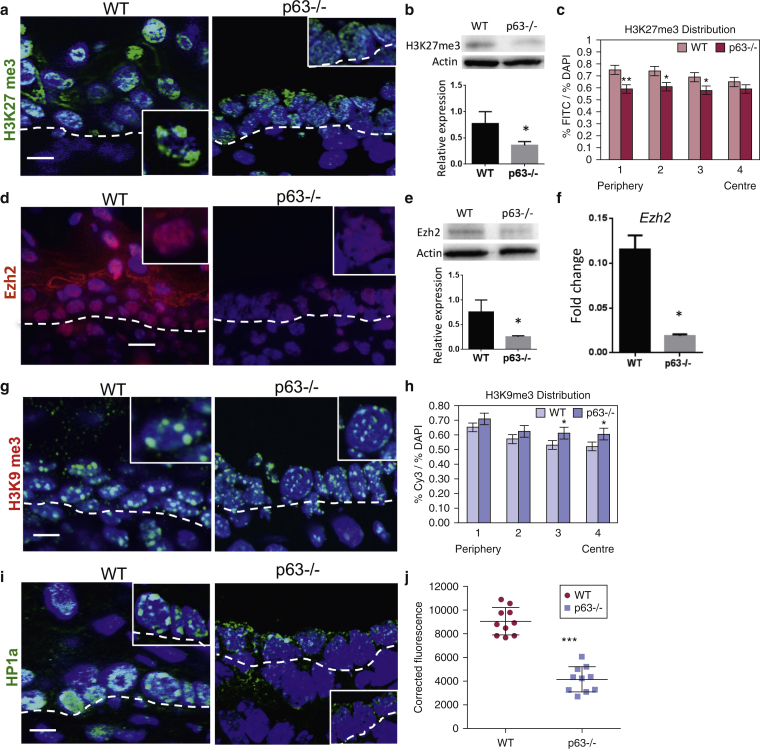
**Alterations in the nuclear distribution of heterochromatin in *p63*^*−/−*^ epidermis.** Immunofluorescence analysis for H3K27me3, Ezh2, H3K9me3, HP1a in the E16.5 skin of WT and *p63*^*−/−*^ mice. Dashed lines depict the dermal-epidermal junction. (**a, b**) Reduced expression of H3K27me3 in p63-null keratinocytes detected by (**a**) immunofluorescent and (**b**) western blot analyses (n = 2, mean ± SD, *P < 0.05). Scale bar = 10 μm. (**c**) Loss of H3K27me3 peripheral distribution in p63-null (one-way ANOVA test, mean ± SEM, *P < 0.05, **P < 0.01. (**d–f**) Significant decrease of (**d, e**) Ezh2 protein and (**f**) transcript expression in p63-null epidermis (n = 2, mean ± SD, *P = 0.05). Scale bar = 10 μm. (**g, h**) Altered distribution pattern of H3K9me3 with a significant increase of its internal distribution (*P = 0.03) in p63-null keratinocytes (mean ± SEM, n = 3). Scale bar = 10 μm. (**i, j**) Marked decrease of HP1a in p63-null epidermis compared with wild-type control (***P < 0.001, mean ± SD, n = 3). Scale bar = 10 μm. ANOVA, analysis of variance; SD, standard deviation; SEM, standard error of the mean; WT, wild-type.

Reduced levels of lamins and other NE proteins alter the distribution of trimethylation on lysine 27 of histone H3 (H3K27me3) and trimethylation on lysine 9 of histone H3 (H3K9me3) histone modifications, established markers of transcriptionally inactive chromatin (Kim and Kim, 2012, Le et al., 2016, Shumaker et al., 2006). Our analysis revealed a global decrease of H3K27me3 in p63-null keratinocytes compared with controls (Figure 4a and b). Although H3K27me3 was significantly enriched at the nuclear periphery in close contacts with the nuclear lamina in WT keratinocytes, it was evenly distributed within the nuclei in p63-null keratinocytes (Figure 4c; Supplementary Figure S5a online). Because polycomb repressive complex 2 is responsible for H3K27me3 deposition in keratinocytes (Bardot et al., 2013, Ezhkova et al., 2009, Ezhkova et al., 2011, Perdigoto et al., 2016), we analyzed expression of its subunits in p63-null mice. Although there were no changes in *Ezh1* and *Eed* expression, Ezh2 and *Suz12* were significantly reduced in the epithelium of *p63*^*−/−*^ mice compared with WT controls (Figure 4d–f; Supplementary Figure S6a online). Furthermore, a polycomb repressive complex 1-dependent histone modification (H2AK119Ub) was reduced in the p63-null epidermis but not in the dermal cells (Supplementary Figure S6b–d). As *Ring1* and *Rnf2*/*Ring1b*, catalytic subunits of polycomb repressive complex 1, were not decreased in the p63-null cells (Supplementary Figure S6e and f), the H2AK119Ub1 reduction was likely caused by the decreased polycomb repressive complex 2 activity in p63-null cells (Schwartz and Pirrotta, 2013, van Kruijsbergen et al., 2015).

Analysis of H3K9me3, a histone modification associated with the pericentromeric heterochromatin, revealed its altered distribution with a significant increase of H3K9me3 foci at the nuclear interior in p63-null keratinocytes compared with controls (Figure 4g and h). Quantification of H3K9me3 foci showed that they were more numerous (approximately 12 foci/nucleus) and smaller in size in p63-null nuclei compared with the controls (approximately 8 foci/nucleus) (Figure 4g; Supplementary Figure S5b online), suggesting that heterochromatin clustering and organization was affected in p63-deficient cells. In line with these observations, we found a reduced expression of HP1α, which interacts with H3K9me3 and is responsible for the pericentromeric heterochromatin organization and clustering (Jones et al., 2000), as well as the loss of its peripheral localization in p63-null nuclei compared with WT controls (Figure 4i and j).

Furthermore, we found a greater reduction and loss of H3K27me3 and H3K9me3 peripheral distribution in cells with abnormal nuclei compared with cells with relatively normal nuclear shape in *p63*^*−/−*^ mice (Supplementary Figure S5c and d online). The latter suggests that there is a direct correlation between nuclear shape alterations and changes in heterochromatin reorganization in *p63*^*−/−*^ mice.

### Keratinocyte-specific gene loci relocate away from the sites of active transcription toward constitutive heterochromatin in *p63*^*−/−*^ mice

Keratin genes are profoundly downregulated in *p63*^*−/−*^ mice (Supplementary Figure S7 online) and clustered in two distinct genomic loci, keratin type I (KtyI) and keratin type II (KtyII), located on mouse chromosomes 11 and 15, respectively (Fessing et al., 2011, Koster et al., 2006, Truong et al., 2006, Viganò et al., 2006).

**Figure 5:**
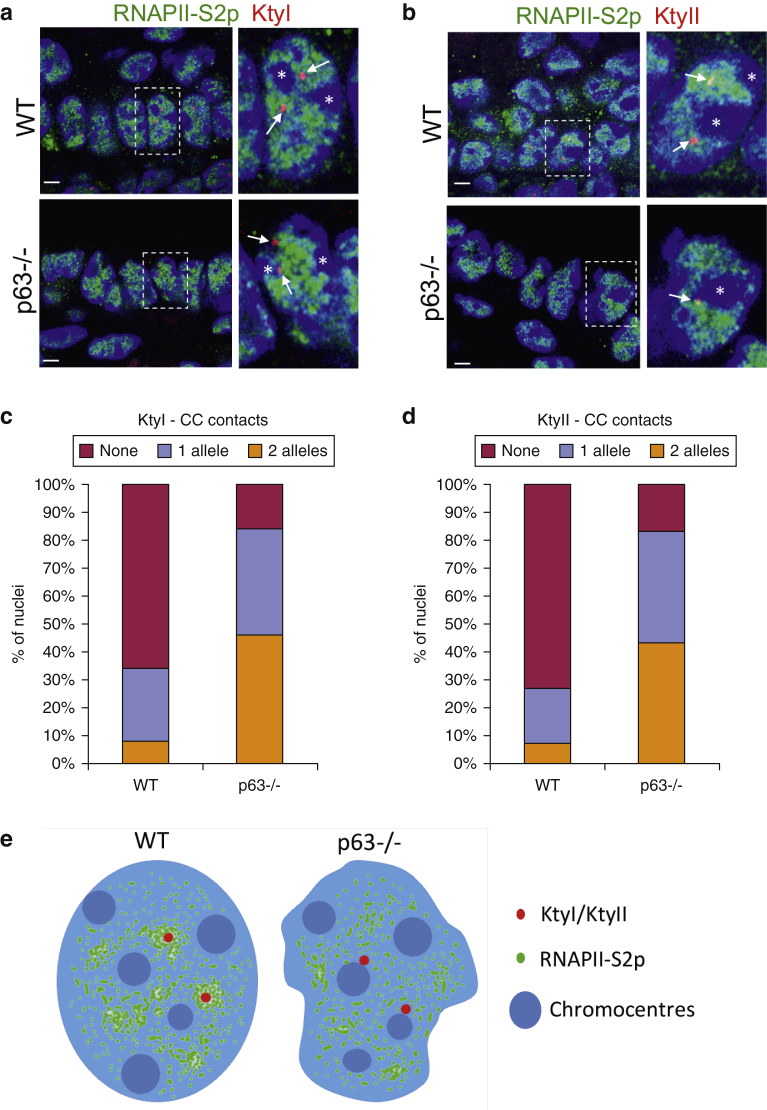
**Relocation of keratinocyte-specific gene loci toward chromocenters in *p63*^*−/−*^ epidermis.** (**a, b**) Multicolor 3D-FISH with probes for (**a**, arrows) KtyI or (**b**, arrows) KtyII followed by immunodetection of pSer2-Pol II in the epidermis of *p63*^*−/−*^ and control mice at E16.5; * labels chromocenters. Scale bars = 5 μm. (**c, d**) p63-null keratinocytes show an increase in the portion of nuclei where both alleles of (**c**) KtyI or (**d**) KtyII are closely associated with the chromocenters (CCs) (n = 50). (**e**) A schematic diagram depicting the position of KtyI and KtyII loci relative to DAPI-dense chromocenters and polymerase II-enriched active site of transcription. 3D-FISH, 3D fluorescence in situ hybridization; KtyI, keratin type I; KtyII, keratin type II; WT, wild-type.

To test whether the alterations in heterochromatin organization affect intranuclear positioning of keratin genes and contribute to their transcriptional silencing in *p63*^*−/−*^ mice, we performed 3D fluorescence in situ hybridization experiments on WT and *p63*^*−/−*^ mouse skin at E16.5 using DNA probes covering KtyI and KtyII loci. The FISH probes were coimmunostained with an antibody against the elongating form of RNA polymerase II phosphorylated at Ser-2, which is enriched in actively transcribed genomic regions (Estaras et al., 2013, Srivastava and Ahn, 2015, Zentner et al., 2011). Both KtyI and KtyII loci were localized in the nuclear interior enriched in RNA polymerase II phosphorylated at Ser-2 in WT cells (Figure 5a and b). In striking contrast, KtyI and KtyII loci in p63-null keratinocytes were found predominantly in RNA polymerase II phosphorylated at Ser-2-depleted sites in close proximity to DAPI-dense chromocenters (CCs), the sites of pericentromeric heterochromatin (Figure 5a and b). Quantification of the KtyI/II loci association with CCs revealed a striking increase in the number of cells where both alleles of keratin loci were in close contact with CCs in *p63*^*−/−*^ keratinocytes compared with WT cells (Figure 5c and d). In contrast, the number of nuclei where no such contacts observed was dramatically reduced in p63-null keratinocytes compared with controls (Figure 5c–e).

The observed data are intriguing, as CCs are known to be the clusters of pericentromeric satellite repeats of chromosomes that cosegregate and comprise the constitutive heterochromatin creating a repressive environment in the nucleus (Politz et al., 2013, Wijchers et al., 2015). Furthermore, CCs are enriched in heterochromatin marks, such as H3K9me2/3 (Supplementary Figure S5e online) (Lienert et al., 2011, Magklara et al., 2011). Together, these data demonstrate that KtyI/II loci relocate to a repressive environment composed of constitutive heterochromatin in p63-null keratinocytes, which highlights a causative link between nuclear shape alterations and heterochromatin redistribution that lead to changes in intranuclear gene positioning and expression of keratinocyte-specific genes in *p63*^*−/−*^ mice.

## Discussion

Here, we show that p63 regulates nuclear shape in epidermal progenitor cells during skin development. Both in vivo and in vitro studies revealed a significant portion of p63-deficient keratinocytes with misshapen nuclei associated with a marked decrease in nuclear lamins and NE-associated proteins. Previous studies showed that murine cells lacking nuclear lamins or cells from patients with Hutchinson-Gilford progeria syndrome, harboring Lamin A mutations, display misshapen nuclei similar to those observed in p63-null keratinocytes (Goldman et al., 2004, Jung et al., 2014, Lammerding et al., 2004, Vergnes et al., 2004, Yang et al., 2011). Together, these observations suggest that reduced expression of Lamin B1 and Lamin A/C may contribute to the defects in nuclear shape observed in *p63*^*−/−*^ mice. However, *Lmnb1* and *Lmna* are likely regulated indirectly by p63, as we could not identify enrichment of p63 at the promoter regions of both genes. Nevertheless, the nuclear lamin reduction may significantly contribute to the skin defects in *p63*^*−/−*^ mice, as the triple lamin knockout mice develop defective epidermal barrier and hypotrophic hair follicles (Jung et al., 2014).

In addition to nuclear lamins, several NE-associated proteins, including plectin, nesprin-3, and Sun1, were significantly downregulated in p63-null keratinocytes. Plectin is a cytoskeletal linker protein of the plakin family that is associated with filamentous actin, IFs, and hemidesmosomal integrins in basal keratinocytes (Nievers et al., 2000, Rezniczek et al., 1998). Plectin is also required for attachment of the nucleus to cytoplasmic IFs via interaction with the linker of nucleoskeleton and cytoskeleton protein nesprin-3 (Ketema et al., 2013, Wilhelmsen et al., 2005). Loss of plectin in keratinocytes reduces keratin IF density around the nucleus and leads to abnormal nuclear morphology. The latter is linked to several skin defects, associated with extremely fragile epidermis and severe skin lesions, including the epidermolysis bullosa complex (Ackerl et al., 2007, Almeida et al., 2015, Gostynska et al., 2015).

Nesprin-3 can uniquely link the NE to the IF network and is also suggested to be involved in maintaining the structural integrity and shape of the nucleus (reviewed in Ketema and Sonnenberg, 2011). Nesprin-3 maintains perinuclear cytoskeleton architecture in endothelial cells and *Zebrafish* epidermal cells (Morgan et al., 2011, Postel et al., 2011). However, the role of nesprin-3 in the homeostasis of mammalian epidermis remains unclear. Loss of nesprin-2 (structurally related to nesprin-3) in human keratinocytes results in variable NE morphological changes from minor NE blebbing to severely misshapen and giant nuclei (Luke et al., 2008). The largest isoforms of *Syne1* and *Syne2* loci termed nesprin-1 and nesprin-2 giant, respectively, interact directly with nesprin-3 via their N-terminal actin binding domains at the outer NM. Collectively, this suggests that nesprin-3 may also regulate the nuclear shape in keratinocytes either via plectin-mediated binding to IFs or by modulating the nesprin-1 and -2 interplay with the cytoskeleton (Lu et al., 2012).

As a part of the linker of nucleoskeleton and cytoskeleton complex, Sun1 is important for localization of nesprins at the outer NM and their interaction with the nuclear lamina (Padmakumar et al., 2005). Sun1-null mice show defects in the formation and positioning of nuclei and cellular dysfunction in several tissues (Ding et al., 2007, Horn et al., 2013, Lei et al., 2009). Sun proteins also play a critical role in the maintenance of epidermal integrity. Downregulations of Sun1 expression along with lamin A/C and emerin were detected in K1/K10-null mice leading to premature nuclear loss during epidermal differentiation (Wallace et al., 2012). Deletion of Sun2, a functional homologue of Sun1, also showed aberrant nuclear position, altered desmosome distribution, and mechanically defective adhesions resulting in defective hair follicle structure and alopecia (Stewart et al., 2015).

Collectively, our data suggest that downregulation of nuclear lamins and NE-associated proteins can lead to the nuclear shape alterations observed in p63-null keratinocytes. To date, it is unclear whether the maintenance of correct nuclear shape by p63 influences p63-dependent gene expression program during epidermal development. Our data provide some answer to the question by revealing that p63 is involved in the control of heterochromatin organization, which is frequently associated with the nuclear periphery in close contacts with nuclear lamins and lamina-associated components (Bank and Gruenbaum, 2011). We showed that p63 deletion in keratinocytes reduces Ezh2 and HP1a expressions and alters the distribution of heterochromatin-associated H3K27me3, H2AK119ub1, and H3K9me3 histone marks suggesting that heterochromatin organization is affected in these cells. In line with these observations, KtyI and KtyII gene loci move away from the RNA polymerase II phosphorylated at Ser-2-enriched sites of active transcription toward the heterochromatin-enriched CCs in p63-null keratinocytes. Collectively, these data raise an intriguing possibility that p63 maintains an active transcriptional environment around the highly expressed keratin genes and prevents heterochromatin redistribution and/or spreading, at least in part, via regulating the expression of NE-associated proteins and controlling a proper nuclear shape. Our finding is also supported by a resent observation that mechanical force-dependent depletion of NE-associated protein emerin alters the H3K9me2/3 and H3K27me3 levels leading to chromatin rearrangements and reduced transcription of lineage-specific genes in human epidermal progenitor cells (Le et al., 2016).

Other p63-depenent mechanisms can also be involved in maintaining the high transcriptional state in keratin loci in epithelial cells. We previously showed that p63 directly regulates the expression of ATP-dependent chromatin remodeler Brg1, which contributes to a developmentally regulated relocation of the epidermal differentiation complex locus toward the transcriptionally active nuclear interior in epidermal progenitor cells (Mardaryev et al., 2014). A similar function for Brg1 was demonstrated in other cell lineages (Christova et al., 2007, Clapier and Cairns, 2009). However, the role of Brg1 in the control of KtyI/II loci nuclear positioning in keratinocytes requires further investigations.

In summary, our data demonstrate a previously unreported role of p63 in coupling the cytoskeleton and nuclear shape regulation with a 3D nuclear organization as an essential part of p63-dependent gene expression program. By regulating expression of NE-associated genes, p63 is involved in maintaining facultative and constitutive heterochromatin organization in epidermal keratinocytes. However, more detailed and genome-wide studies are required to address to what degree the nuclear shape regulation and heterochromatin organization contribute to the p63-dependent gene expression program keratinocytes. In particular, DNA adenine methyltransferase identification analysis in p63-null keratinocytes will be important to identify changes in the lamina-associated domains that dynamically associate with lamina and contain many developmentally regulated and/or tissue-specific genes (Lin et al., 2012, Meister et al., 2010, Peric-Hupkes et al., 2010). Future research in this direction will shed some more light on the complex p63-dependent regulatory network that controls epidermal development and its maintenance, as well as provides a mechanistic insight into the pathological conditions with underlined p63 dysfunction.

## Materials and Methods

### Experimental animals and tissue collection

Animal studies were performed in accordance with protocols approved by the UK Home Office Project License. C57Bl/6 mice were purchased from Charles River. p63-null and WT embryos were obtained by breeding p63^+/−^ animals from Jackson Laboratories. Skin samples were collected from mice at distinct days of embryonic and postnatal development (E16.5 and P0.5), as described previously (Botchkarev et al., 1999b, Sharov et al., 2003, Sharov et al., 2005). Genotyping of mice was performed using PCR, as recommended by the supplier. For each developmental stage, six to seven samples were collected. Tissue samples were covered in Tissue-Tek O.C.T. Compound (VWR, Lutterworth, UK), snap-frozen in liquid nitrogen, and stored in −80 °C.

### Immunofluorescence, 3D fluorescence in situ hybridization, and image analysis

Histological sections of quick frozen E16.5 *p63*^*−/−*^ and age-matched WT embryos were fixed in 4% paraformaldehyde and stained with specific primary and secondary antibodies (Supplementary Table S1 online), as described previously (Botchkarev et al., 1999a, Fessing et al., 2006, Sharov et al., 2003, Sharov et al., 2005). Nuclei of basal keratinocytes from *p63*^*−/−*^ mice, p63 siRNA-treated PMKs, and corresponding control cells were counted using ImageJ software and percentages of nuclei with altered morphology were calculated in Microsoft Excel spreadsheets. Circularity values were obtained using “Circularity” ImageJ plugin (http://rsb.info.nih.gov/ij/plugins/circularity.html).

For the analysis of the distribution of histone modifications, the nuclear geometric center of nuclei (n = 35) from both WT and *p63*^*−/−*^ embryos was found and lines were drawn from the nuclear center to the nuclear periphery. To cover the entire nuclear surface, eight radial lines have been drawn throughout the nuclear center and values of fluorescence intensity have been collected using ImageJ plugin “Plot Profile.” Measurements from each line have been subsequently divided and grouped into four different shells, averaged and normalized to the mean percentage of DAPI signal in that shell.

Immunofluorescence intensity was determined using ImageJ software, as described previously (Mardaryev et al., 2016). Briefly, regions of interest were selected within WT or *p63*^*−/−*^ epidermis and dermis, and the corrected values of total cell fluorescence (CTCF) were calculated for each selected areas using the following formula: CTCF = Integrated Density − (Area of selected cell × Mean fluorescence of background readings). For pairwise comparisons, a two-tailed *t*-test (α = 0.05) was employed.

DNA probe preparation and 3D fluorescence in situ hybridization analysis were performed as described previously (Fessing et al., 2011, Mardaryev et al., 2014). 3D images were collected using a Zeiss LSM510 confocal microscope. Images were processed and analyzed using ImageJ. DAPI-enriched CCs were considered to be within the vicinity of gene loci when the corresponding fluorescent signals were found to at least partially overlap.

### Laser capture microdissection and quantitative real-time reverse transcriptase-PCR analysis

Laser capture microdissection of whole mouse epidermis of E16.5 *p63*^*−/−*^ and age-matched WT controls was performed followed by RNA extraction and amplification, as published before (Fessing et al., 2011). Total RNA was extracted using the ReliaPrep RNA Cell Miniprep System kit (Promega, Southampton, UK), followed by two rounds of amplification using the RiboAmp RNA amplification kit (Life Technologies, Waltham, MA). For quantitative real-time reverse transcriptase-PCR analysis, RNA was retrotranscribed into cDNA and specific primers were designed using the Beacon Designer software (Supplementary Table S2 online; PREMIER Biosoft International, Palo Alto, CA). Real-time PCR was performed using SYBER-Green Master Mix (Life Technologies) on the StepOne Plus system (Life Technologies). Differences between samples were calculated based on the Ct (ΔΔCt) method and normalized to *Gapdh*. Data from triplicates were pooled, mean ± standard deviation was calculated, and statistical analysis was performed using unpaired Student’s *t*-test.

### Cell culture and siRNA transfection

PMKs were isolated from newborn 2- to 3-day-old C57Bl/6 mice, as described previously (Lewis et al., 2014, Mardaryev et al., 2016). PMKs were grown in EMEM calcium-free medium (Lonza, Wolverhampton, UK) with supplements (0.05 mM calcium, 4% fetal bovine serum, 0.4 μg/ml hydrocortisone, 5 μg/ml insulin, 10 mg/ml epidermal growth factor (EGF), 10^−10^ M cholera toxin, 2 × 10^−9^ T3, 2 mM l-glutamine, 100 U/ml penicillin, and 100 μg/ml streptomycin) at 33 °C, 8% CO_2_ until 60–70% confluent. PMKs were transfected with 100 nM p63siRNA or control siRNA using Lipofectamin 2000 (Life Technologies).

### ChIP-qPCR assay

ChIP assay was performed using epidermal keratinocytes isolated from newborn mouse skin with a p63 antibody or IgG control, as published previously (Mardaryev et al., 2011). Briefly, cross-linked DNA after sonication was precipitated with 5 μg of anti-p63 antibody or nonimmune goat IgG (Vector Laboratories, Burlingame, CA) overnight at 4 °C. Purified ChIPed DNA was amplified with gene specific primers (Supplementary Table S3 online). ChiIP-qPCR data from triplicates were pooled, mean ± standard deviation was calculated, and statistical analysis was performed using Student’s *t*-test.

## Conflict of Interest

The authors state no conflict of interest.
