# Supplementary material for: p63 Transcription Factor Regulates Nuclear Shape and Expression of Nuclear Envelope-Associated Genes in Epidermal Keratinocytes
Source: J Invest Dermatol. 2017 Oct;137(10):2157–67. doi: 10.1016/j.jid.2017.05.013 (PMC5610935; doi:10.1016/j.jid.2017.05.013)
Supplement: Supplementary Data [file mmc1.pdf]

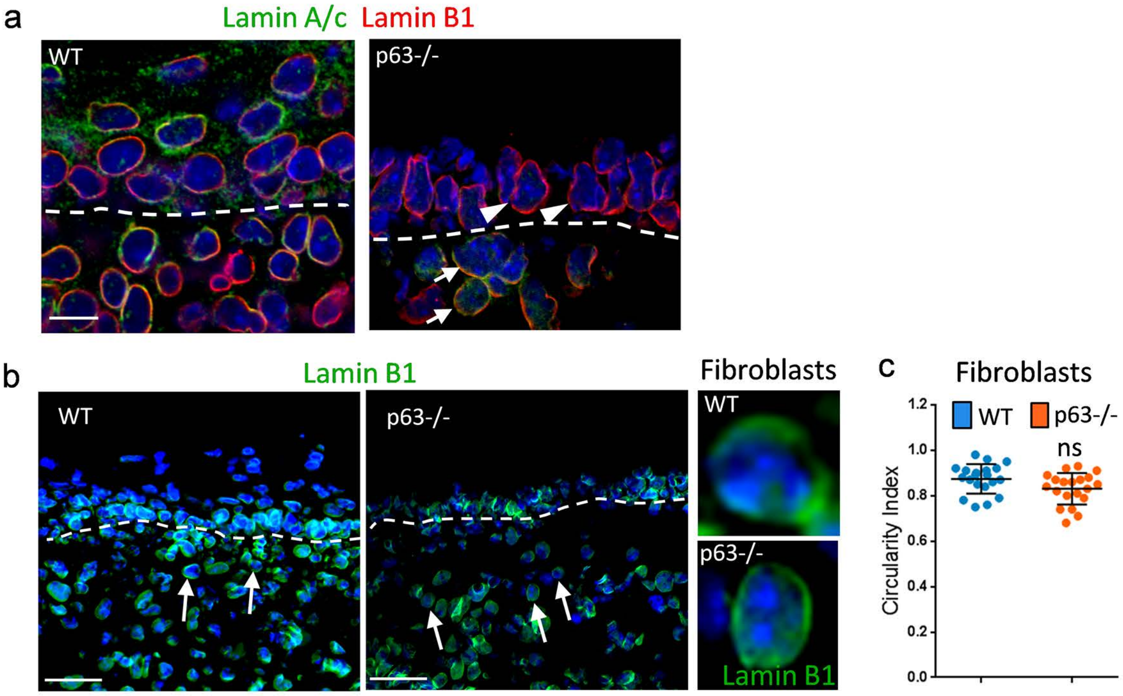

Figure S1. Expression of lamins and nuclear circularity index of dermal fibroblasts in p63-/- mice.

a – Lamin A/C is markedly down-regulated in the p63-/- epidermal cells (arrowheads), but its expression is unchanged in dermal fibroblasts (arrows);

b, c – Expression of Lamin B1 and nuclear circularity index are not changed in p63-/- dermal fibroblasts. Scale bars,10um (a) and 50 um (b),

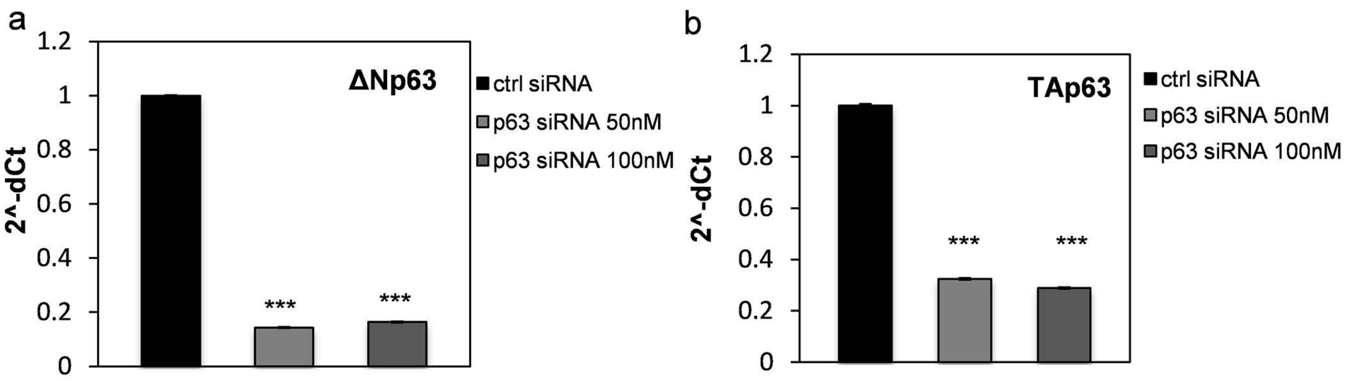

Figure S2. qRT-PCR analysis of ΔNp63 and TAp63 isoforms mRNA expression after p63 siRNA knock-down in Primary Mouse Keratinocytes.

a - qRT PCR analysis performed using primers specific for ΔNp63 isoform, showing significant reduction (p-value<0.0001) of this isoform after p63 knock-down in Primary Mouse Keratinocytes using p63 siRNAs in comparison to control siRNAs.

b - qRT PCR analysis performed using primers specific for TAp63, showing significant reduction (p-value<0.0001) of this isoform after p63 knock-down in Primary Mouse Keratinocytes using p63 siRNAs in comparison to control siRNAs.

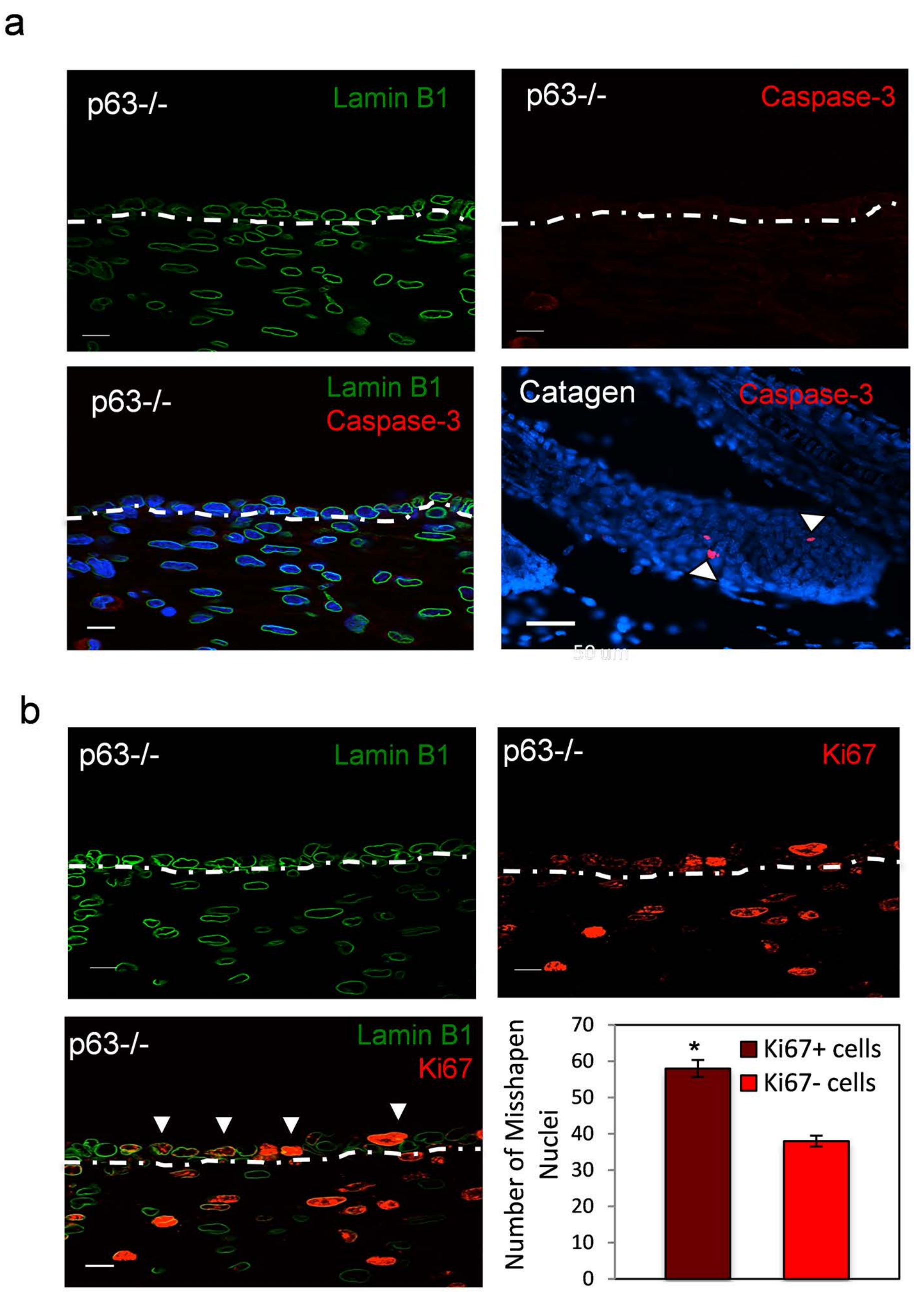

Figure S3. Analysis of apoptosis and proliferation in p63-null keratinocytes nuclei displaying altered shape. Immuno-fluorescence analysis for Caspase-3 and Ki67 in E16.5 skin of $p63^{-/-}$ mice. Dashed lines depict dermal-epidermal junction.

a - Analysis of apoptotic cells using caspase-3 specific antibody (red), showing that all keratinocytes with misshapen nuclei were negative for caspase-3. Scale bars, 10 um. Catagen hair follicles have been used as a positive control for caspase-3 antibody specificity (Scale bars, 50 um);

b - Analysis of proliferation using Ki67-specific antibody (red, arrowheads) of keratinocytes with misshapen nuclei, revealing that the majority of cells with altered nuclear morphology were positive for Ki67 (Student's t-test, mean+/-SEM, *p<0.05, n=3). Scale bars, 10 um.

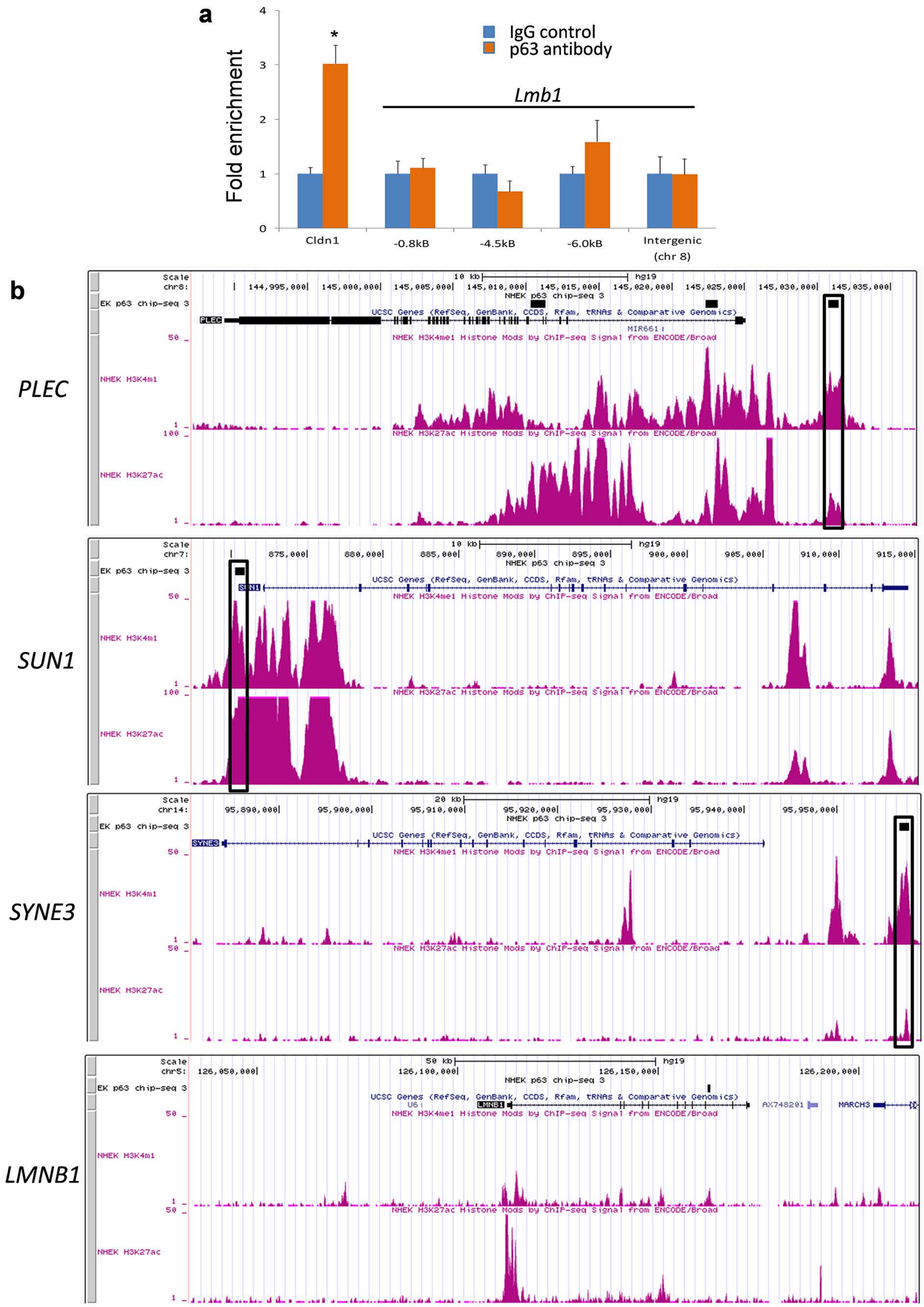

Figure S4. p63 is not enriched on *Lmb1* in Primary Mouse Keratinocytes.

a - Several regions (indicated) within the *Lmnb1* promoter analysed by qPCR after immunoprecipitation of chromatin with anti-p63 antibody. Error bars represent SD, and four independent experiments were run in triplicates; $p<0.05$;

b –ChIP-seq tracks for p63 and histone modifications (H3K4me1 and H3K27a) in NHEKs. p63 (black bar) is co-enriched with the histones marks (pink signals) in the promoter and enhancer regions (up to 10kb away from TSS) of *PLEC, SUN1* and *SYNE3* genes (black frame). Note, p63 does not bind to *LMNB1* gene in the promoter and distant enhancer regions (up to 50 kB away from TSS) in NHEKs.

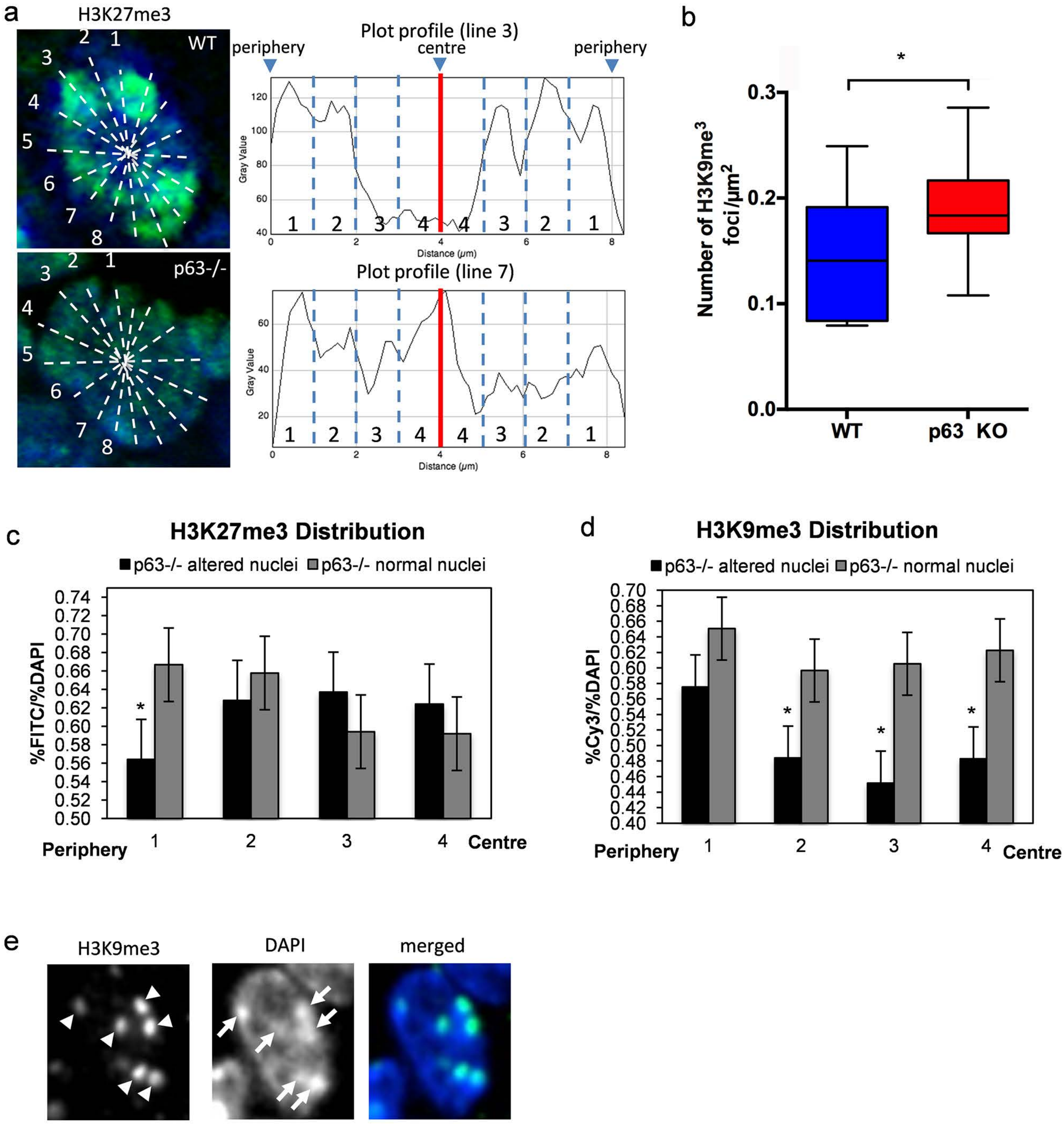

Figure S5. Alterations in the distribution patterns of repressive histone marks, H3K27me3 and H3K9me3, in p63-null keratinocytes.

a - Analyses of H3K27me3 distribution in wt vs p63-/- keratinocyte nuclei. Note a loss of peripheral enrichment of H2K27me3 in p63-/- cells.

b – Increased number of H3K9me3 foci in epidermal cells of p63-null mice (normalised to area of the middle section of each nuclei); box plot with mean and min and max values, Student's t-test, n=3, *p < 0.05.

c, d - Analysis for the distribution of repressive histone modifications H3K27me3 and H3K9me3 in p63-null keratinocytes nuclei with normal and altered shape, showing significant (p-value=0.05) decrease of H3K27me3 at the periphery of nuclei with altered shape in comparison to nuclei with normal shape (c). H3K9me3 is significantly reduced (p-value=0.05) in p63-null nuclei with altered shape in comparison to normal shaped keratinocytes nuclei was shown (d).

e – Co-localisation of H3K9me3 (arrowheads) with DAPI-dense chromocenters (arrows) in basal keratinocytes.

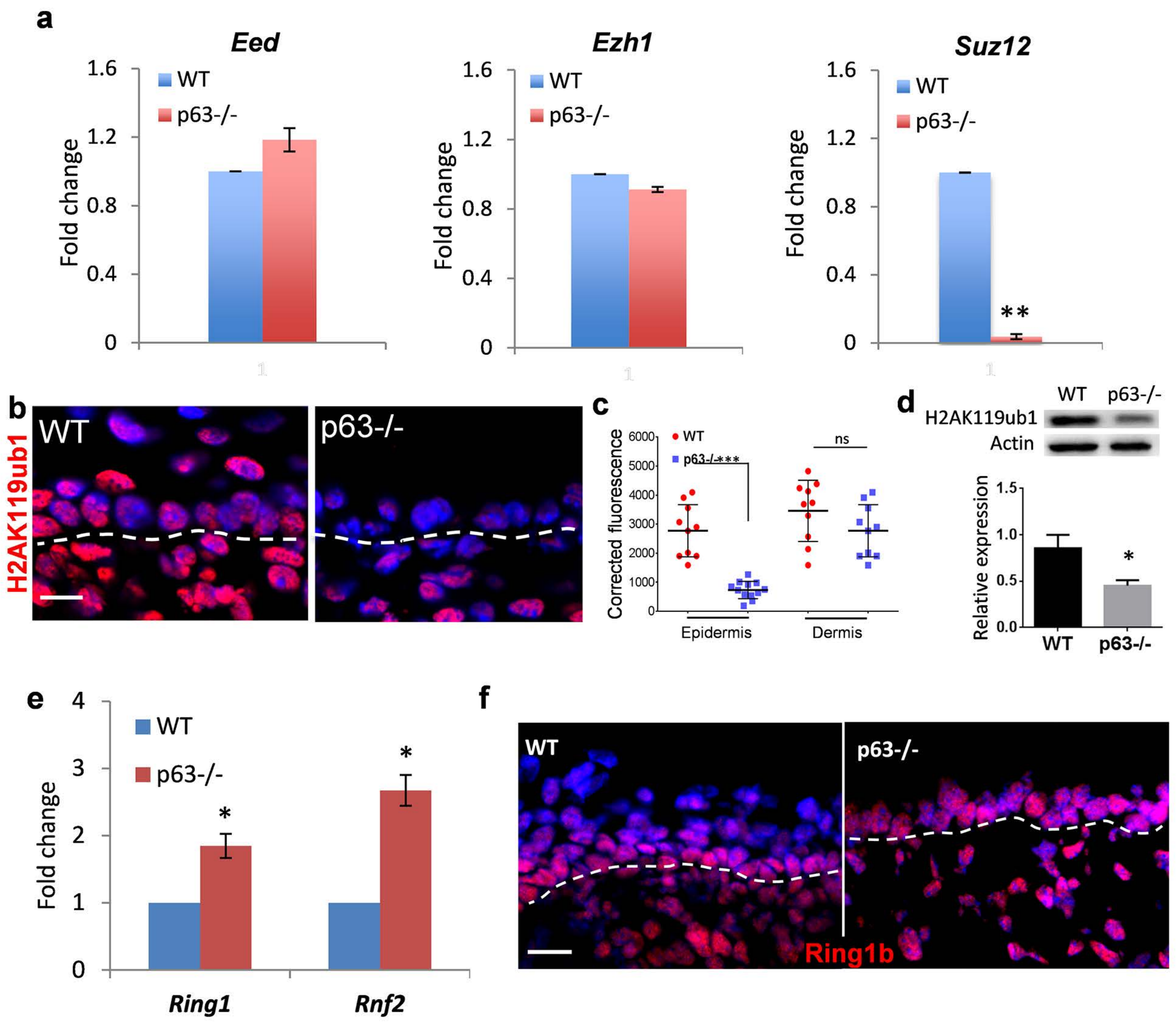

Figure S6 Expression of PRC1/2 core subunits in p63-/- epidermis.

a - RT-qPCR analyses of Ezh1, Eed and Suz12 in p63-/- keratinocytes vs controls. Students t-test, n=2, mean +/- sem; ** <0.001

b-d– Immunofluorescent detection (b), quantification (c) and western blot analysis of H2AK119Ub1 expression in WT and p63-/- skin. H2AK119ub1 is markedly reduced in the p63-/- epidermis, while its expression is unchanged in the dermal cells (**p*<0.05, ***p<0.001, mean+/- SD, n=3). Scale bar, 10 um.

e - Expression of *Ring1* and *Rnf2/Ring1b* transcripts in p63-null keratinocytes. Students t-test, n=2, mean +/- sem; **p* <0.05;

f - Ring1b protein expression is not affected in p63-/- epithelium; scale bar, 25um; dashed lines show epidermal-dermal junction.

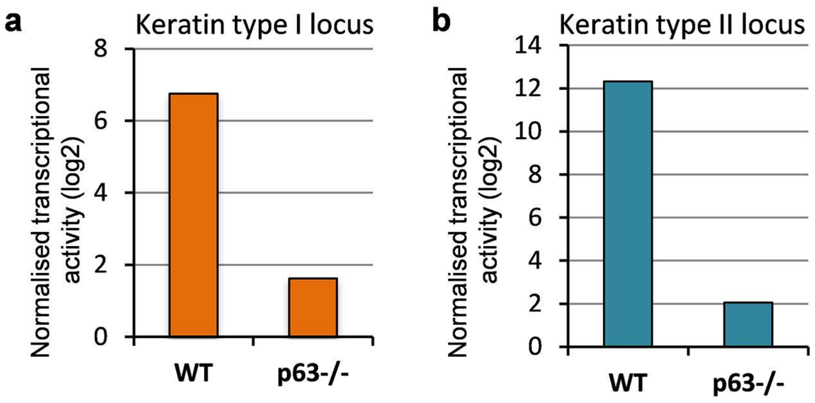

Figure S7. Decreased gene expression and intranuclear location of KtyI (A) and KtyII (B) loci in p63-/- keratinocytes.

a-b - Sum of background-corrected Agilent microarray expression values normalised to number of genes per locus.

Supplementary Data

Materials and Methods

Western blot analysis

Embryonic skin from WT and p63-/- mice and primary mouse keratinocytes were lysed with CelLytic M lysis buffer (Sigma-Aldrich) containing protease inhibitor cocktail (Roche Applied Bioscience, Rotkreuz, Switzerland) to prevent protein degradation. Protein concentration was determined by Bradford assay (ThermoFisher Scientific). Total protein was incubated in 4X Loading buffer (200mM Tris Base, pH6.8, 40% v/v glycerol, 8% w/v SDS, 0.04% w/v bromophenol blue and 20% v/v beta-mercaptoethanol) for 5min at 95°C. 30µg of total protein was separated according to their molecular weight using 10-12.5% SDS-PAGE. Proteins were blotted onto Nitrocellulose membrane (GELifesciences, USA) and membrane blocked in 3% w/v dried semi-skimmed milk in 1XTBST (10mM Tris Base pH7.2, 0.15M NaCl and 0.1% v/v Tween-20) for 1 hour on a shaker at 4°C. Membrane was probed with α-EZH2 (Santa Cruz, USA) at 1:2000, α-H3K27me3 (Cell signalling, UK) at 1:1000 and α-H2AK119ub1 (Cell signalling, UK) at 1:1000 in 1XTBST containing 3% w/v dried semi-skimmed milk overnight at 4°C with gentle shaking. HRP-conjugated α-rabbit or α-mouse antibodies (Cell signalling) were used at 1:2000 in 1xTBST for 1 hour at 4°C with gentle shaking. Membranes were incubated in Super Signal West Femto substrate (Thermo Scientific) for 5 minutes at room temperature and protein bands were detected using Chemidoc imaging system (Bio-Rad). For a repeat Western blot for loading control, membranes were stripped at room temperature for 30 minutes using stripping buffer (25mM Glycine, 1% SDS, 0.02% Sodium Azide and 0.1% β-Mercaptoethanol) and blocked with 3% dried semi-skimmed milk in 1XTBST for 30 minutes prior to probing with α-actin

antibody(Abcam) at 1:2000 and HRP-conjugated α-mouse antibody (Cell signalling) at 1:2000.

ChIP-seq analysis

The ChIP-seq peaks and signal tracks for H3K4me1 and H3K27ac modifications (hg19 build) in NHEK were obtained from ENCODE consortium and p63 ChIP-Seq peaks track in keratinocytes (hg18 build) were obtained from GEO database (accession number GSE17611). The UCSC Genome Browser Lift Over tool was used to convert p63 ChIP-Seq peaks genome coordinates to hg19 build. The H3K4me1, H3K27ac, p63 tracks were uploaded to UCSC Genome Browser for data visualisation and analysis.

Statistics

At least, three WT and p63-/- embryos were used for each experiment. All experiments were performed in triplicate and repeated independently at least two times. Two-tailed unpaired Student's t-test or one-way analysis of variances (ANOVA) were used to detect differences using GraphPad PRISM ver. 6 for Windows (GraphPad Software, San Diego, CA). Results are expressed as mean ± standard deviation or mean ± standard error of the mean as indicated. A $p < 0.05$ was considered significant.

Table S1. List of primary antibodies

Antigen	Host	Application	Dilution/amount	Manufacturer
LaminB1	Goat	IF	1-500	Iakowos Karakesisoglou'Lab
Plectin	Goat	IF	1-100	Iakowos Karakesisoglou'Lab
Lamin A/C	Rabbit	IF	1-200	Santa Cruz Biotechnology
Ezh2	Rabbit	IF	1-100	Abcam
	Rabbit	WB	1-2000	Santa Cruz, USA
H3K27me3	Rabbit	IF	1-100	Abcam
	Rabbit	WB	1-1000	Cell Signaling
H3K9me3	Rabbit	IF	1-100	Abcam
Sun1	Rabbit	IF	1-100	Santa Cruz Biotechnology
Hp1α	Rabbit	IF	1-100	Abcam
Nesprin-3	Rabbit	IF	1-100	Santa Cruz Biotechnology
Caspase-3	Rabbit	IF	1-100	Abcam
Ki67	Rabbit	IF	1-100	Abcam
H2AK119ub	Rabbit	IF	1-100	Active Motif
	Rabbit	WB	1-1000	Cell Signaling
p63 (4A4)	Mouse	ChIP	4 ug	Santa Cruz Biotechnology
IgG control	Mouse	ChiP	4 ug	Active Motif

Table S2. List of primers for quantitative RT-PCR

Gene symbol	Accession Number	Forward Oligo	Reverse Oligo
Lmnb1	**NM_010721**	GAGGAGGAGGAGGAG	CAAGTTCACATAATGCCACAG
Plec	**NM_201389**	CGCCATTACCAGCAGTTAC	GCACAGTCCGAGTCTCAC
Lmna	**NM_001002011**	CCACTCATCCCAGTCTCAG	CTTCCTCTACCGCCACAC
Syne3	**NM_001042699**	AGACAGCCACAGAGGATG	TTCTTCAGTTGAGTTTGTAACC
Sun1	**NM_001256115**	TACTCGTCGGATGCTCTG	CCTGCTGGTGCTAATGTG
Ezh1	**NM_007970**	CAGCCAGGATATGTTGATGC	GGATCATCTCTTCTTCACCG
Ezh1	**NM_007971.2**	TGATGATGATGATGACGATGATGG	TCCGAGGTGGGCAAGTTTC
Ring1	**NM_009066**	GATGGTACAGAGATTGCGGTTT	CCTATGCAGGCACTCCTTGG
Rnf2	**NM_011277**	GAGTTACAACGAACACCTCAGG	CAATCCGCGCAAAACCGATG

Table S3. List of primers for PCR analysis after ChIP

Gene symbol	Forward Oligo	Reverse Oligo
Plectin 1c promoter (chr15:76232952-76233245)	TCTGGTTCCCTGCTCTACAAAGTC	CCCCACTCCCCAAACAGGTC
Syne3 promoter (chr12:105010406-105010569)	GAGAGGAGATGGACATTCTTGG	GAGCGACACAGGCAACAG
Sun1 promoter (chr5:139196701-139196830)	ACTCTGCCTGCCATCTTCTTCTG	TTGTTGTTGTGGTCATCGG
Claudin1 promoter (chr16:26372802-26373008)	TGGAAGCATCCCTTGTTTTC	TTGCTGTCCTCTCTGGGTCT
Intergenic regions (chr8:73659729-73659868)	AAGGGGCCTCTGCTTAAAAA	AGAGCTCCATGGCAGGTAGA
